# The Surprising Diversity of UV‐Induced Mutations

**DOI:** 10.1002/ggn2.202300205

**Published:** 2024-03-07

**Authors:** Marian F. Laughery, Hannah E. Wilson, Allysa Sewell, Scott Stevison, John J. Wyrick

**Affiliations:** ^1^ School of Molecular Biosciences Washington State University Pullman WA 99164 USA

**Keywords:** atypical photoproducts, DNA repair, reactive oxygen species, skin cancer, ultraviolet light, UVA

## Abstract

Ultraviolet (UV) light is the most pervasive environmental mutagen and the primary cause of skin cancer. Genome sequencing of melanomas and other skin cancers has revealed that the vast majority of somatic mutations in these tumors are cytosine‐to‐thymine (C>T) substitutions in dipyrimidine sequences, which, together with tandem CC>TT substitutions, comprise the canonical UV mutation “signature”. These mutation classes are caused by DNA damage directly induced by UV absorption, namely cyclobutane pyrimidine dimers (CPDs) or 6‐4 pyrimidine‐pyrimidone photoproducts (6‐4PP), which form between neighboring pyrimidine bases. However, many of the key driver mutations in melanoma do not fit this mutation signature, but instead are caused by T>A, T>C, C>A, or AC>TT substitutions, frequently occurring in non‐dipyrimidine sequence contexts. This article describes recent studies indicating that UV light causes a more diverse spectrum of mutations than previously appreciated, including many of the mutation classes observed in melanoma driver mutations. Potential mechanisms for these diverse mutation signatures are discussed, including UV‐induced pyrimidine‐purine photoproducts and indirect DNA damage induced by UVA light. Finally, the article reviews recent findings indicating that human DNA polymerase eta normally suppresses these non‐canonical UV mutation classes, which can potentially explain why canonical C>T substitutions predominate in human skin cancers.

## Graphical abstract

UV predominately causes C>T mutations in melanoma, but many driver mutations do not fit this signature. This review discusses recent genome sequencing studies indicating that UV produces a more diverse spectrum of mutations than previously suspected, but these are suppressed by the activity of human DNA polymerase eta. Mutagenic mechanisms are also discussed, including atypical photoproducts and oxidative damage.

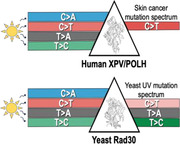

## 1 Introduction

Exposure to ultraviolet (UV) light is the leading risk factor in the development of melanoma and non‐melanoma skin cancers. Whole genome‐sequencing of these tumors has revealed that as many as 90% of all UV‐induced somatic mutations are cytosine to thymine (C>T) substitutions in dipyrimidine sequences.^[^ ^1^, ^2^, ^3^ ^]^ UV‐induced DNA lesions such as cyclobutane pyrimidine dimers (CPDs) and 6‐4 pyrimidine‐pyrimidone photoproducts (6‐4PPs), which form between neighboring pyrimidine bases (i.e., dipyrimidine sequences), are presumed to be the primary cause of these mutations.^[^ ^4^, ^5^ ^]^ Skin cancer genomes also have abundant tandem CC>TT substitutions, which account for as many as ≈5% of mutations.^[^ ^1^, ^3^ ^]^ Together, these comprise the primary elements of the UV mutation signature.^[^ ^4^, ^6^ ^]^

In addition to this unique mutation pattern, a second characteristic feature of UV signature mutations in skin cancers is that they occur less frequently on the transcribed strand (TS) of genes than the non‐transcribed strand (NTS) or intergenic DNA, a feature known as transcriptional asymmetry.^[^ ^7^, ^8^ ^]^ Transcriptional asymmetry is due to more rapid repair of CPDs, and potentially other forms of UV damage, on the TS by the transcription coupled‐nucleotide excision repair (TC‐NER) pathway.^[^ ^9^, ^10^, ^11^, ^12^ ^]^ The remainder of the genome (i.e., NTS and intergenic DNA) is repaired by the global genomic‐nucleotide excision repair (GG‐NER) pathway,^[^ ^13^ ^]^ which is less efficient than TC‐NER.

While most passenger mutations in melanoma are C>T (and CC>TT) substitutions in dipyrimidine sequences, most driver mutations, including mutations occurring in the *BRAF* and *NRAS* oncogenes, do not fit this UV signature.^[^ ^2^, ^3^ ^]^ For example, *BRAF* V600E is the most frequently observed oncogenic mutation among melanoma patients, but this mutation is a T>A substitution in a non‐dipyrimidine sequence context (i.e., GTG>GAG).^[^ ^2^, ^14^, ^15^ ^]^ Similarly, the three most frequent *NRAS* mutations (i.e., *NRAS* Q61R, Q61K, Q61L) are due to T>C, C>A, and T>A substitutions, respectively, in the *NRAS* Q61 (i.e., CAA/TTG) codon.^[^ ^3^, ^16^, ^17^ ^]^ Given that tumor sequencing is by nature a retrospective snapshot of the complex process of carcinogenesis, the resulting mutation landscape is likely shaped not only by distinct mutational processes, but also by selection. Hence, to what extent these driver mutations are caused by solar UV exposure and the potential mechanism involved is unknown.

While medium wavelength UVB light (i.e., 280–315 nm) is thought to primarily induce mutations by forming CPDs and 6‐4PPs in DNA, previous studies have hinted that UV light can also cause rare atypical photoproducts, such as thymine‐adenine (TA) photoproducts.^[^ ^18^, ^19^, ^20^, ^21^ ^]^ However, until recently, to what extent these atypical photoproducts contribute to UV mutagenesis was unclear. Moreover, long wavelength UVA light (i.e., 315–400 nm) is also thought to cause mutations, but whether this is primarily due to direct DNA damage in the form of CPDs, or through indirect DNA damage that is mediated by reactive oxygen species (ROS) generated after UVA absorption by photosensitizing compounds, remains a long‐standing question in the field. Finally, although sunlight is the major source of UV exposure, artificial sources of UV light, including tanning beds and even nail dryers used in nail salons (predominately emitting long wavelength UVA light), may also cause mutations and contribute to skin cancer risk.

## 2 New Methods for Characterizing UV‐Induced Mutations

Prior studies of UV‐induced DNA mutagenesis in *E. coli*, yeast and mammalian cells commonly relied on mutation reporter genes, in which mutations that inactivate the reporter gene (e.g., *CAN1* or *URA3* in yeast and *HPRT* or bacterial *SupF* in mammalian cells) can be selected for using canavanine (*CAN1*), 5‐fluoroorotic acid (*URA3*), or 6‐thioguanine (*HPRT*), and the resulting inactivating mutations can be sequenced. These studies have provided evidence that UV induces not only abundant signature mutations (i.e., C>T and CC>TT in dipyrimidine contexts) most likely derived from CPDs and/or 6‐4PPs, but also a variety of less common mutations whose origins were largely unclear (e.g.,^[^ ^22^, ^23^, ^24^, ^25^, ^26^, ^27^, ^28^, ^29^ ^]^). While these and other studies made important contributions to our understanding of UV mutagenesis, these mutation reporters have limitations due to the typically small (<100) number of mutations identified, all of which occurred in a single genomic context, and potential biases introduced due to the finite number of mutation possibilities that can inactivate the reporter gene.

**Figure 1:**
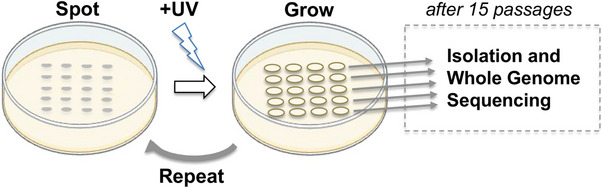
Passaging assay for UV mutagenesis in yeast. Yeast cells are spotted onto plates containing rich media, UV irradiated, and allowed to grow in standard culture condition. The resulting cell spots are then diluted, re‐spotted onto fresh plates, and subjected to the same process for a total of fifteen passages. Cells from each spot are struck for isolation and whole genome sequencing is performed on genomic DNA extracted from a clonal isolate obtained from each spot.

In the past few years, we and others have used genome sequencing methods to analyze genome‐wide patterns of UV mutagenesis.^[^ ^17^, ^30^, ^31^, ^32^, ^33^, ^34^, ^35^ ^]^ Our method involves exposing separate patches of diploid yeast (*Saccharomyces cerevisiae*) to sublethal doses of UV radiation, and allowing the cells to regrow under normal culture conditions between each exposure (**Figure** **1**). This process is repeated multiple times (i.e., typically 15x) to allow for the accumulation of UV‐induced mutations in the genome over the course of the passaging experiment. The resulting mutations are then identified by whole genome sequencing of DNA extracted from a single clonal isolate derived from each patch (Figure 1).^[^ ^17^, ^32^, ^33^ ^]^

There are many benefits to using yeast as a model organism for studying UV mutagenesis. The small genome of yeast, coupled with the ease of culturing and genetically modifying its genome, make this organism ideal for using whole genome sequencing to identify UV‐induced mutations from replicate experiments with diverse genetic backgrounds. For example, we typically perform whole genome sequencing of ≈20–40 independent clonal isolates for each genotype studied.^[^ ^17^, ^32^, ^33^ ^]^ Yeast are also more resistant to UV radiation than mammalian cells, allowing them to be irradiated at higher UV doses, yet they have similar repair pathways (e.g., GG‐NER and TC‐NER) as human cells, making them valuable models of UV damage, repair, and mutagenesis. Inactivation of pathways such as global genomic DNA repair (GG‐NER) can clarify the roles of these pathways in the repair of specific mutation types and sequence contexts,^[^ ^17^, ^33^ ^]^ and such mutants are readily available in yeast. Additionally, the lack of selection in the passaging method, in part due to the use of diploid strains to minimize the potential impacts of mutation‐induced gene inactivation, makes the accumulation of mutations via passaging unbiased, unlike mutation data that is obtained from reporter gene experiments. This feature, in conjunction with the large number of mutations that are obtained from repeatedly exposing yeast to UV light, enables more detailed characterization and statistical analysis of the resulting UV‐induced mutation classes, which can give insight into the origins of these mutations.^[^ ^17^, ^32^, ^33^ ^]^ Finally, in contrast to the relatively high levels of endogenous background mutations that can occur in mammalian cells grown in culture,^[^ ^30^ ^]^ yeast experience very low background levels of mutagenesis. This feature is particularly useful for discerning the potential role of UV in inducing less abundant, atypical mutation classes such as T>A, T>C, and C>A mutations (see below). In short, yeast passaging assays are a valuable tool for directly observing the complete spectra of mutations that arise from a mutagenic source and for studying the roles that repair enzymes and translesion DNA polymerases play in their occurrence.

## 3 A Diverse Spectrum of UVB‐Induced Mutations

Although UVB light (∼280‐315 nm) comprises only a small proportion (≈5–10%) of the UV radiation from the sun that reaches the Earth, it is thought to be the primary cause of sunlight‐induced DNA lesions and mutations. UVB exposure induces CPDs and 6‐4PPs, which form between neighboring dipyrimidine sequences (i.e., TT, TC, CT, and CC).^[^ ^27^ ^]^ These lesions are thought to give rise to the C>T and CC>TT mutations that comprise the canonical UV mutation signature and are the primary mutation classes observed in human skin cancers.^[^ ^1^, ^2^, ^3^, ^4^, ^5^, ^8^ ^]^

**Figure 2:**
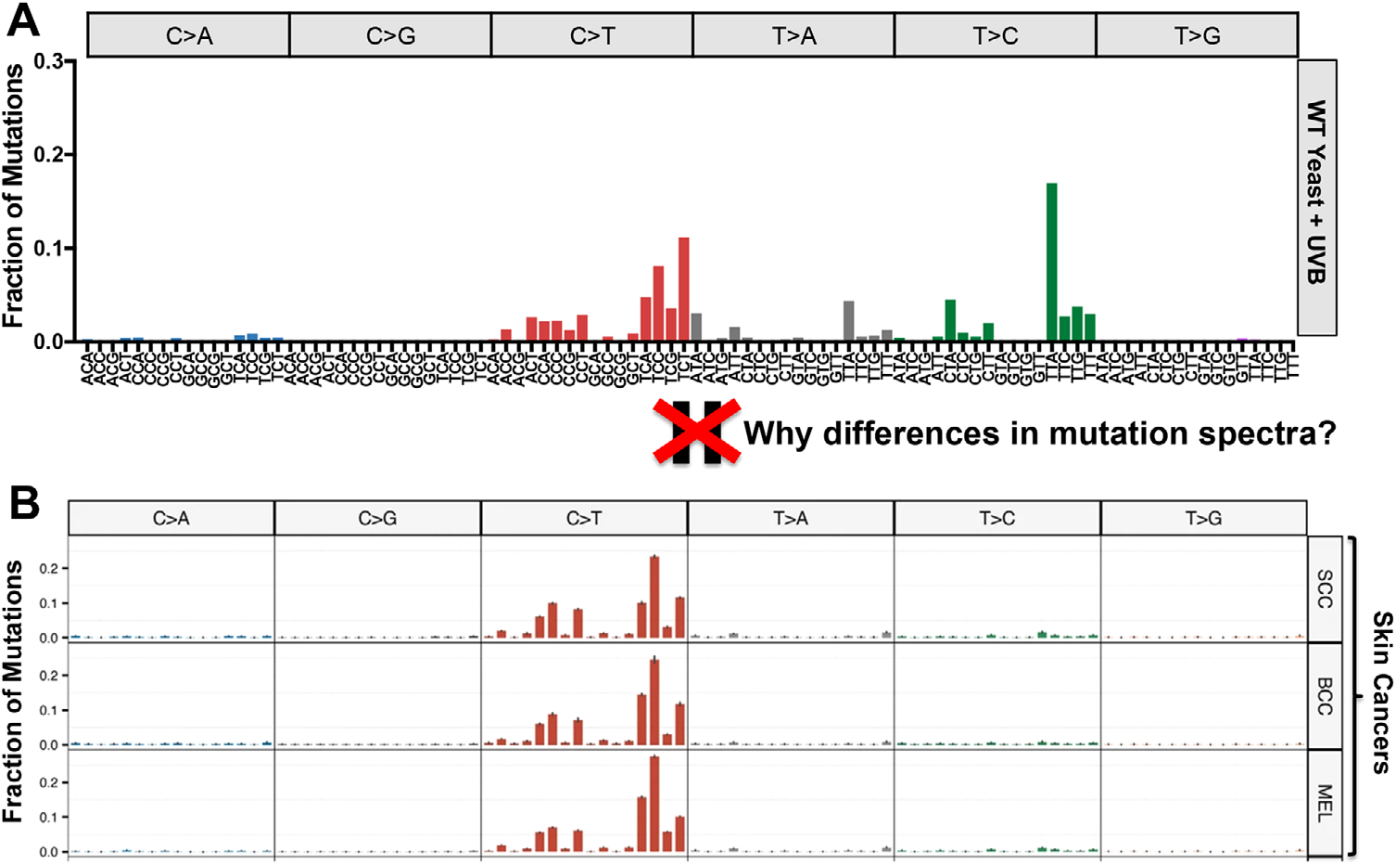
Differing mutation spectra of UV‐exposed yeast and human skin cancers. A) Mutation spectrum derived from whole genome sequencing data from UVB‐exposed wild‐type yeast cells. The fraction of total mutations associated with each mutation class and trinucleotide context is depicted. Data are from.^[^ ^33^ ^]^ B) Mutation spectra derived from whole genome sequencing of sporadic human skin cancers, including squamous cell carcinomas (SCC), basal cell carcinomas (BCC), and melanomas (MEL). The fraction of total mutations associated with each mutation class and tri‐nucleotide context is depicted. Figure is reproduced and adapted under terms of the Creative Commons Attribution 4.0 International License.^[^ ^34^ ^]^ 2023, The Authors, published by Nature Communications.

However, a recent analysis of a compendium of UVB‐induced mutations in yeast revealed a surprisingly diverse mutation spectrum.^[^ ^33^ ^]^ This study identified UVB‐induced mutations by performing whole genome sequencing on multiple yeast isolates that had been exposed to 15 doses of UVB light (see section 2 above). Altogether, ≈6500 single base substitutions were identified in UVB‐exposed wild‐type (WT) yeast isolates, the vast majority of which were likely caused by UVB light, since unexposed control isolates had ≈100‐fold fewer mutations.^[^ ^33^ ^]^ Of these, only 42% of UV‐induced mutations were canonical C>T substitutions in dipyrimidine sequences (**Figure** **2**A). In contrast, 80–90% of single base substitutions in human skin cancers are C>T substitutions in dipyrimidine sequences (e.g., Figure 2B).^[^ ^1^, ^3^, ^34^ ^]^ In UVB‐exposed yeast, there were also abundant T>C (≈36%) and T>A (≈14%) substitutions, together comprising half of all UVB‐induced mutations (Figure 2A).^[^ ^33^ ^]^ In contrast, T>C and T>A substitutions, while present in the skin‐cancer associated COSMIC signatures SBS7c and SBS7d,^[^ ^3^, ^16^, ^36^, ^37^ ^]^ are generally relatively rare in melanomas (MEL), squamous cell carcinomas (SCC) and basal cell carcinomas (BCC; see Figure 2B).^[^ ^1^, ^3^, ^34^ ^]^ In UVB‐exposed yeast, the most abundant mutation type is T>C substitutions in a TTA sequence context (i.e., TTA>TCA mutations; see Figure 2A), comprising 17% of all UVB‐induced mutations in yeast,^[^ ^33^ ^]^ yet these mutations are rare in human skin cancers. Very similar UV mutation classes (i.e., C>T, T>C, and T>A substitutions) are observed in yeast repeatedly exposed to UVC (≈254 nm) light.^[^ ^17^ ^]^ In contrast, whole genome sequencing of human induced pluripotent stem (iPS) cells exposed to simulated solar radiation only revealed an enrichment of C>T (and CC>TT) substitutions at dipyrimidine sequences,^[^ ^30^ ^]^ consistent with the mutation spectrum of human skin cancers.

**Figure 3:**
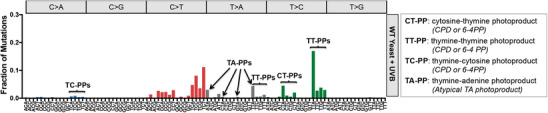
Non‐canonical UV‐induced mutations are enriched in UVB passaged yeast. Mutation spectrum from UVB‐irradiated yeast (same as Figure 2A) is depicted.^[^ ^33^ ^]^ The putative lesion responsible for each mutation class is indicated. Mutations occurring in dipyrimidine contexts (i.e., CT, TT, and TC) likely originate from CPDs or 6‐4 PPs, which are the two most prevalent UV lesions, which can potentially explain UV‐induced C>A, T>C, and a subset of T>A mutations. In contrast, mutations occurring in NTA contexts likely originate from atypical thymine‐adenine photoproducts and yield T>A/A>T mutations.

These striking differences in UV mutation spectra raise the question of what molecular mechanism(s) are responsible for T>A, T>C, and other unusual mutation classes in UV‐exposed yeast. It is important to note that previous studies using mutation reporter genes observed similar UV‐induced mutation classes, including T>A and T>C substitutions,^[^ ^22^, ^24^, ^25^ ^]^ but the molecular mechanisms involved were unclear. Importantly, the vastly greater number of UVB‐induced mutations identified by whole genome sequencing has provided important clues as to their origin.^[^ ^17^, ^33^ ^]^ First, many of these mutation classes, including nearly all T>C and certain T>A (e.g., TTT>TAT, TTC>TAC, and TTG>TAG) mutation classes are specifically enriched at dipyrimidine sequences, suggesting that they may be caused by canonical UV‐induced photoproducts (PPs), namely CPDs and 6‐4PPs (**Figure** **3**). Second, these mutation classes displayed transcriptional asymmetry, with fewer mutations on the transcribed strand of genes.^[^ ^33^ ^]^ Third, whole genome sequencing of *rad16*∆ GG‐NER deficient cells repeatedly exposed to UVB light revealed that these mutation classes were elevated in the repair‐deficient cells, suggesting they arise from UV lesions repaired by the NER pathway.^[^ ^17^, ^33^ ^]^ For these reasons, the working hypothesis is that these non‐canonical mutations are caused by canonical UV photoproducts (i.e., CPDs or 6‐4PPs; see Figure 3).^[^ ^17^, ^33^ ^]^ It will be important in future studies to determine whether CPDs or 6‐4PPs are primarily responsible for inducing these classes of T>A and T>C substitutions.

Interestingly, UVB passaging and sequencing assays in yeast also revealed abundant T>A mutations associated with NTA sequence contexts (i.e., ATA>AAA, CTA>CAA, GTA>GAA, and TTA>TAA; see Figure 3).^[^ ^33^ ^]^ Many of these contexts do not contain a dipyrimidine (i.e., ATA and GTA), indicating that they are not caused by CPDs or 6‐4PPs. Moreover, analysis of transcriptional asymmetry indicated that these mutation classes are elevated on the transcribed strand of yeast genes relative to the non‐transcribed strand,^[^ ^17^, ^33^ ^]^ an asymmetry that is opposite that of other UV‐induced mutations. The simplest explanation for these findings is that the T>A mutation is actually an A>T substitution associated with a thymine‐adenine (TA) lesion on the opposite strand (i.e., TAT>TTT, TAG>TTG, TAC>TTC, and TAA>TTA). Notably, these mutation classes are also elevated in repair‐deficient *rad16*∆ cells, indicating that they are likely caused by a helix‐distorting UV photoproduct.

Based on this evidence, the current model is that these A>T substitutions are caused by atypical UV‐induced thymine‐adenine (TA) photoproducts. UV‐induced TA photoproducts were identified four decades ago^[^ ^18^ ^]^ and have since been characterized by multiple groups.^[^ ^17^, ^19^, ^20^, ^21^, ^38^, ^39^, ^40^, ^41^ ^]^ Studies of the mutational properties of a TA photoproduct in *E. coli* yielded a similar mutational signature (i.e., TA>TT substitutions) to what was detected in UV‐irradiated yeast.^[^ ^19^ ^]^ Recently, our group has developed a new sequencing method known as UVDE‐seq to specifically detect TA photoproducts,^[^ ^17^, ^40^, ^41^ ^]^ and found that they are specifically elevated at TAT and TAA sequences. This sequence specificity of TA photoproduct formation can potentially explain why A>T substitutions are enriched in these same sequence contexts (i.e., T>A in ATA or TTA sequence contexts; see Figure 3).^[^ ^39^ ^]^ Taken together, these studies indicate that UVB light also causes non‐canonical mutation classes (i.e., A>T substitutions) by inducing atypical thymine‐adenine photoproducts.^[^ ^17^, ^33^, ^39^, ^41^ ^]^ It will be important in future studies to determine to what extent these atypical UV photoproducts are responsible for inducing driver mutations in melanoma.

## 4 Species‐Specific Differences in UV Mutation Spectra are Caused in Part by DNA Polymerase Eta

A key question is why the abundant T>A and T>C mutation classes observed in UV‐irradiated yeast (Figure 2A) are largely absent from human skin cancers (Figure 2B). New insight into this question has recently been provided by a study characterizing mutation patterns in skin cancers arising in patients with Xeroderma Pigmentosum (XP).^[^ ^34^ ^]^ XP patients are extremely sensitive to the damaging effects of UV exposure and especially prone to skin cancers due to an inherited genetic deficiency in one of eight XP genes.^[^ ^27^, ^42^, ^43^ ^]^ Seven of these (*XPA* to *XPG*) encode proteins involved in NER, rendering the cells in XP patients unable to efficiently repair UV photoproducts. The eighth gene, which is mutated in XP variant (XP‐V) patients, is not involved in NER, and *XPV^−/^* ^‐^ cells still efficiently repair UV photoproducts. Instead, these patients have a genetic deficiency in DNA polymerase eta (*POLH*/*XPV*), which is a translesion synthesis (TLS) DNA polymerase involved in the bypass of UV‐induced photoproducts and other DNA lesions.^[^ ^44^, ^45^, ^46^, ^47^, ^48^ ^]^

**Figure 4:**
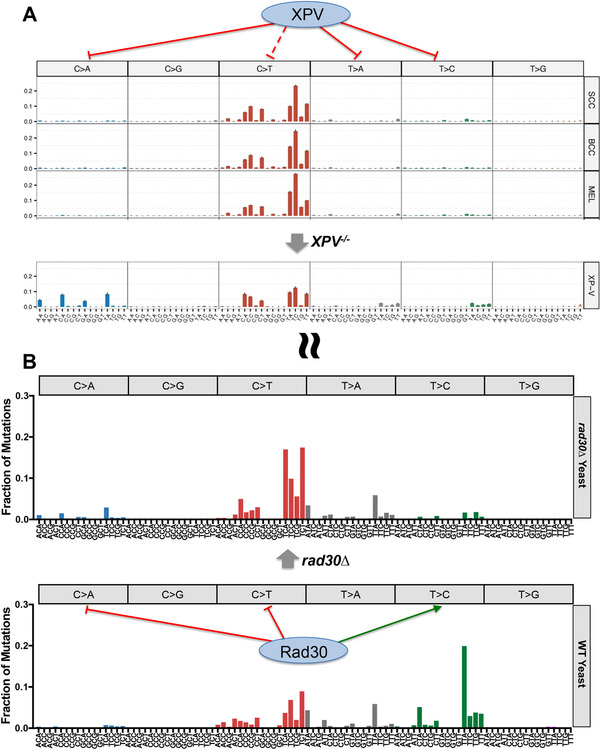
Mutation spectra of human *XPV^−/−^* tumors and yeast rad30∆ mutants show greater similarity. A) Mutation spectra of whole genome sequencing of sporadic skin cancers (top panel; see Figure 2 legend for more details) versus *XPV^−/−^* tumors (bottom panel) reveals increased proportion of C>A, T>A, and T>C substitutions in *XPV* mutant tumors. This effect is primarily reflected in 3′ TT dinucleotide sequences for many of the T>A and T>C substitutions, suggesting an important role for XPV/POLH in error‐free bypass of UV‐induced TT photoproducts. C>A substitutions were primarily noted in NCA sequence contexts and were highly enriched in *XPV^−/−^* tumors. Transcriptional asymmetry analysis (not depicted) indicates that these mutations were actually TG>TT substitutions, potentially originating from a thymine‐guanine (TG) photoproduct. Figure is reproduced and adapted under terms of the Creative Commons Attribution 4.0 International License.^[^ ^34^ ^]^ 2023, The Authors, published by Nature Communications. B) Mutation spectra derived from whole genome sequencing of UVC‐exposed WT yeast (top panel) and *rad30∆* deletion (bottom panel) passaging isolates. Similar to *XPV^−/−^* tumors, yeast data reflects an increase in C>A mutations in NCA sequence contexts in the *rad30*∆ mutant. Transcriptional asymmetry analysis (not depicted) CA>AA substitutions are likely TG>TT substitutions. Conversely, *rad30*∆ in yeast also caused a decreased frequency of T>C substitutions in TT dipyrimidines, suggesting an error‐prone bypass of TT photoproducts by Rad30 in yeast. Further, loss of Rad30 in yeast had no effect on T>A substitutions, suggesting yeast Rad30 may not be able to bypass TA photoproducts in an error‐free manner. Both POLH and Rad30 seem to serve a protective effect against C>T substitutions, particularly in the 3′ position of dipyrimidines. Data from WT and *rad30*∆ mutant yeast exposed to 15 doses of UVC light are depicted.^[32]^

Whole genome sequencing of skin cancers arising in *XPV^−/−^* patients revealed a strikingly different mutation spectra than that seen in sporadic skin cancer cases (**Figure** **4**A).^[^ ^34^ ^]^ These *XPV^−/−^* tumors not only had significantly elevated mutation densities relative to sporadic skin cancers, but among the mutation classes that were most highly elevated in *XPV^−/−^* tumors were T>C, T>A, and especially C>A substitutions. The T>C mutations (and some of the T>A mutation classes) primarily occurred at the 3′ position of TT dinucleotides (e.g., TTA>TCA, TTC>TCC, etc., see Figure 4A).^[^ ^34^ ^]^ Interestingly, T>A/C mutations in TTN contexts showed strand asymmetry, indicating that many of these mutations were caused by lesions repaired by NER, such as CPDs or 6‐4PPs. This mutagenic pattern was recreated in a *POLH* knockout cell line exposed to UVC light, indicating that POLH/XPV is important for error‐free bypass of UV‐induced TT photoproducts (i.e., CPDs or 6‐4PPs).^[^ ^34^ ^]^ In cells lacking POLH/XPV, these TT photoproducts are likely bypassed in an error‐prone manner by an alternative TLS polymerase, such as DNA polymerase iota or kappa.^[^ ^49^, ^50^, ^51^, ^52^, ^53^, ^54^, ^55^ ^]^

*XPV^−/−^* tumors also showed elevated T>A substitutions in an NTA sequence context (i.e., ATA>AAA, CTA>CAA, GTA>GAA, and TTA>TAA). Moreover, this mutation class was found to have the opposite transcriptional asymmetry of UV signature mutations (i.e., enrichment of T>A substitutions on the transcribed strand of genes). These findings led the authors to hypothesize that these T>A mutation classes were in fact A>T substitutions caused by atypical thymine‐adenine photoproducts,^[^ ^34^ ^]^ consistent with whole genome sequencing data from UV‐irradiated yeast.^[^ ^17^ ^]^ Taken together, these data suggest that POLH/XPV is also important for error‐free bypass of UV‐induced TA photoproducts in human cells.

Notably, the most enriched class of mutations were C>A substitutions, which comprised 27% of all somatic mutations in the *XPV^−/−^* tumors.^[^ ^34^ ^]^ These substitutions showed a striking sequence context bias, being primarily enriched in NCA sequences (i.e., ACA>AAA, CCA>CAA, GCA>GAA, and TCA>TAA; see Figure 4A). Analysis of transcriptional asymmetry indicated that they were enriched on the transcribed strand of genes, indicating that the mutations were actually TG>TT substitutions, potentially arising from a thymine‐guanine (TG) photoproduct.^[^ ^34^ ^]^ In support of this hypothesis, whole genome (or exome) sequencing of human cells lacking *POLH/XPV* and exposed to UVA or UVC light also revealed abundant TG>TT substitutions.^[^ ^34^, ^56^ ^]^ While a UV‐induced TG photoproduct has never been described in the literature, it could resemble the well‐characterized thymine‐adenine photoproduct.^[^ ^34^ ^]^ Alternatively, these mutations could reflect mutagenic bypass of an oxidized guanine lesion induced by UV exposure, which may form preferentially when situated immediately 3′ of a thymine base.

A separate study performed a similar analysis of UV‐induced mutations in yeast cells lacking DNA polymerase eta (*rad30*∆).^[^ ^32^ ^]^ Whole genome sequencing of *rad30*∆ diploid yeast exposed to 15 doses of UV light (see section 2) revealed a significantly altered mutation spectra relative to UV‐irradiated WT cells (Figure 4B). Similar to *XPV^−/−^* tumors, *rad30*∆ mutant yeast showed elevated C>A substitutions, again primarily associated with NCA sequence contexts.^[^ ^32^ ^]^ These CA>AA substitutions were also enriched on the transcribed strands of yeast genes, indicating that they are actually TG>TT substitutions occurring on the opposite DNA strand. Taken together, these findings suggest that both yeast and human DNA polymerase eta prevent UV‐induced G>T substitutions, potentially by performing error‐free bypass of a putative TG photoproduct or an oxidized guanine base.

In contrast, loss of DNA polymerase eta in yeast had very different impacts on other non‐canonical UV mutation classes. For example, while *XPV^−/−^* tumors had elevated T>C substitutions at the 3′ position of TT dipyrimidines,^[^ ^34^ ^]^ deletion of *RAD30* in yeast resulted in a decreased frequency of this mutation class (Figure 4B).^[^ ^32^ ^]^ These results suggest that while human DNA polymerase eta protects against T>C substitutions in TT sequences, presumably by performing error‐free bypass of TT photoproducts (i.e., CPDs and 6‐4PPs), yeast Rad30 performs error‐prone bypass of TT photoproducts, thereby promoting UV‐induced T>C substitutions (Figure 4). These results can be potentially explained by a previous study suggesting that yeast Rad30 tends to insert a guanine base opposite the 3′ position of a TT 6‐4PP, thereby causing T>C substitutions.^[^ ^57^ ^]^

While loss of human DNA polymerase eta resulted in an increase in A>T substitutions associated with presumptive TA photoproducts,^[^ ^34^ ^]^ loss of yeast DNA polymerase eta had no effect on this mutation class (Figure 4).^[^ ^32^ ^]^ These results suggest that human DNA polymerase eta is able to perform error‐free bypass of TA photoproducts, while yeast DNA polymerase eta lacks this activity. Finally, both yeast and human DNA polymerase eta appear to protect against UV‐induced C>T substitutions, particularly at the 3′ position of dipyrimidines, although due to the enrichment of other UV‐induced mutation classes in *XPV^−/−^* tumors, there is a decrease in the proportion of C>T substitutions among all somatic mutations in these tumors (Figure 4).

Comparison of the mutation spectra of *XPV^−/−^* tumors (Figure 4A, bottom panel) and UV‐irradiated *rad30*∆ yeast (Figure 4B, top panel) reveals much greater similarity than the mutation spectra of sporadic skin cancer and UV‐irradiated WT yeast, in which DNA polymerase eta is active. These results suggest the intriguing model that species‐specific differences in the activity of DNA polymerase eta are in part responsible for the striking differences in UV mutation spectra between yeast and human cells. In other words, these studies indicate that UV exposure in human cells has the same potential for causing a diverse spectrum of non‐canonical UV mutation classes (e.g., T>A, T>C, etc.), as is observed in yeast, but in human cells this diversity is suppressed by the action of human DNA polymerase eta. This activity of human DNA polymerase eta may be particularly important to prevent skin carcinogenesis, since many of these substitution types are found in melanoma driver genes.^[^ ^2^, ^3^ ^]^

## 5 Mutation Spectrum of UVA Light

The spectrum of mutations that result from UVA radiation (315–400 nm) is somewhat distinct from that of UVB light. DNA bases absorb the longer UVA wavelengths much less efficiently than UVB and this yields substantially reduced CPD levels.^[^ ^58^, ^59^, ^60^ ^]^ Additionally, UVA can cause indirect DNA damage, which occurs when UVA light is absorbed by photosensitizing compounds to yield reactive oxygen species (ROS) that can subsequently damage DNA, although the exact mechanism remains unknown. These ROS yield oxidized bases such as 8‐oxoguanine,^[^ ^23^, ^58^, ^61^ ^]^ which frequently give rise to G>T substitutions. A large body of literature suggests that UVA can induce mutations either through canonical CPD lesions or by inducing oxidative damage, the relative frequency of which depends on many factors, including the particular species and/or cell types being studied.^[^ ^62^ ^]^

Recently, three studies have used whole genome sequencing methods to characterize the mutation spectra of UVA light. One of these irradiated yeast with 15 exposures of a high dose (200 kJ m^−2^) of UVA light. Despite this extensive exposure to UVA, there was only ≈3‐fold induction of mutations in wild type yeast relative to unirradiated controls.^[^ ^33^ ^]^ Significant enrichment of C>A and C>G mutations was observed in UVA passaged yeast, whereas no significant enrichment was observed in UV signature mutations (i.e., C>T in dipyrimidines). Deletion of the gene encoding the DNA repair enzyme Ogg1 (8‐oxoguanine glycosylase) resulted in ∼10‐fold increase in mutations relative to both unirradiated control cells and wild type UV‐irradiated cells, and nearly all of the resulting mutations were C>A/G>T substitutions.^[^ ^33^ ^]^ These data, in combination with strand‐specific mutation reporters, indicated that UVA‐induced guanine oxidation is the primary culprit responsible for UVA‐induced mutations in yeast,^[^ ^33^ ^]^ consistent with a previous report.^[^ ^23^ ^]^

A second study used whole genome sequencing to characterize UVA‐induced mutations in a genetically modified human retinal pigment epithelial (RPE‐1) cell line lacking the *TP53* gene.^[^ ^34^ ^]^ They found that UVA exposure resulted in a ≈2‐fold increase in mutations relative to untreated cells, the most frequent mutation classes being C>T substitutions, primarily in dipyrimidine sequences, and C>A/G>T substitutions. Notably, they found that deletion of *XPV/POLH* in these cells resulted in a significant increase in UVA‐induced mutations (≈7‐fold), which showed a distinct mutation spectrum, including enriched T>A, T>C, and TG>TT substitutions.^[^ ^34^ ^]^ A previous whole exome sequencing study also found that UVA induces C>T substitutions in dipyrimidine sequences in *XPV/POLH* deficient cells.^[^ ^56^ ^]^ Taken together, these findings indicate that DNA polymerase eta also suppresses many UVA‐induced mutation classes.

Finally, a third study examined mutations arising from exposure to UVA light in nail salons.^[^ ^63^ ^]^ Many of these nail salons use UVA‐emitting nail dryers, exposure to which has been potentially linked to skin cancer.^[^ ^63^, ^64^, ^65^ ^]^ This study observed a strong induction of ROS and an absence of CPDs or 6‐4PPs in both mouse and human primary cells following acute and chronic (i.e., three consecutive exposures) to UVA‐emitting nail dryers.^[^ ^63^ ^]^ They found that both chronic and acute UVA exposure from nail dryers caused small but significant increases in mutations in both mouse and human cells, with the most abundant class typically being C>A/G>T substitutions. In summary, this study indicates that non‐solar sources of UVA light can be both DNA damaging and mutagenic.^[^ ^63^ ^]^

## 6 Conclusions and Future Directions

**Figure 5:**
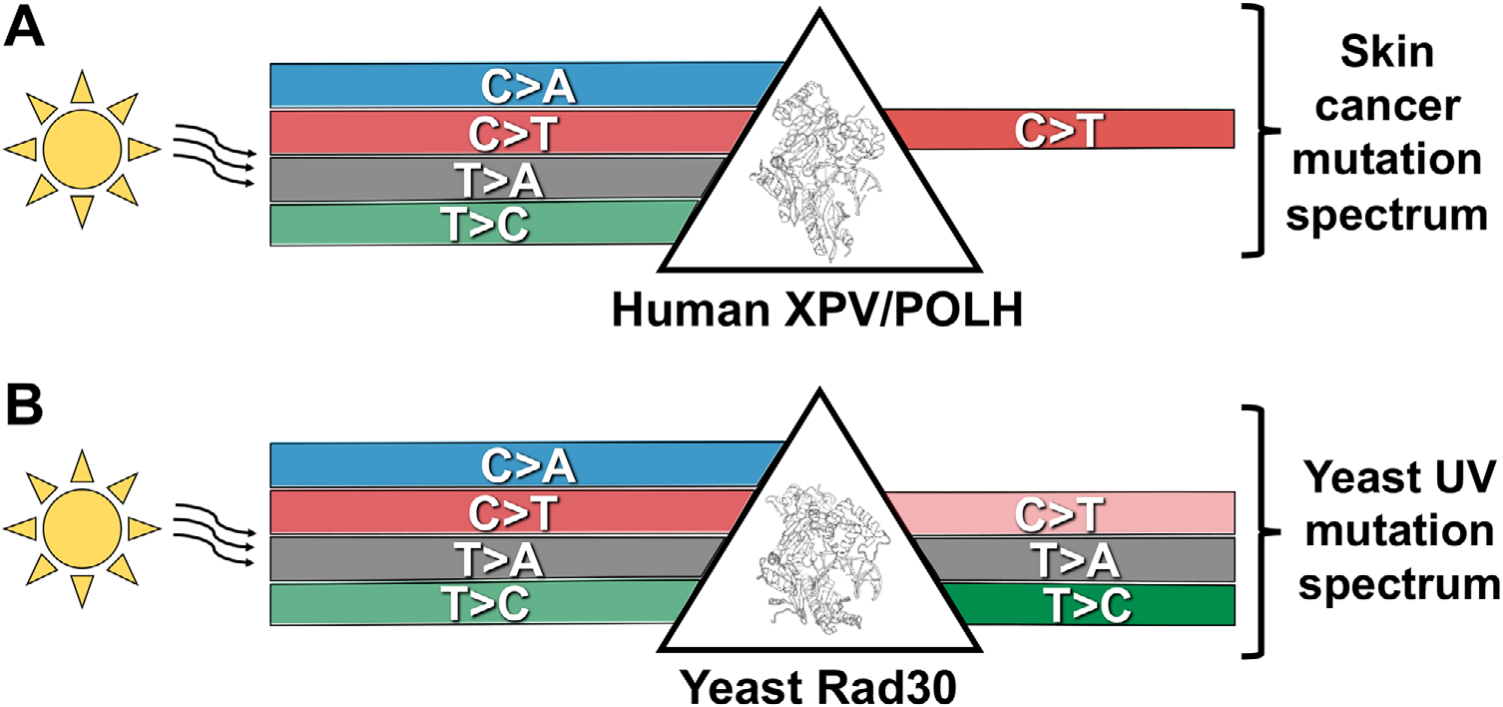
Model of how species‐specific differences in DNA polymerase eta impact UV mutation spectra in yeast and human cells. A) Human *XPV/POLH* suppresses UV‐induced C>A, T>A, and T>C mutations due to error‐free bypass of the causative lesions. Note, while UV‐induced C>T mutations comprise the vast majority of the mutation spectra in skin cancers, C>A, T>A, and T>C mutations do occur at low frequency, even though they are not depicted in model. Structure of human POLH from PDB ID: 4J9S and visualized using Pymol. B) Yeast Rad30 does not suppress UV‐induced T>A mutations and stimulates UV‐induced T>C mutations, likely due to error‐prone bypass of 6‐4PP or CPD lesions. Yeast Rad30 does suppress UV‐induced C>A and, to a lesser extent, C>T mutations. Structure of yeast Rad30 from PDB ID: 3MFH and visualized using Pymol.

While it is well‐known that UV exposure induces a largely monochromatic mutation spectrum in human skin cancers, characterized by C>T (and CC>TT) substitutions in dipyrimidine sequences,^[^ ^3^, ^4^, ^5^, ^66^ ^]^ recent studies have challenged this simple paradigm. Whole genome sequencing of UV‐irradiated yeast cells has revealed a much more diverse spectrum of UV mutations, including C>A, T>A, and T>C substitutions,^[^ ^17^, ^33^ ^]^ consistent with reports from previous mutation reporter studies in yeast.^[^ ^22^, ^24^, ^25^ ^]^ Notably, many of these same mutation classes are enriched in human cells or skin cancers deficient for the XPV/POLH translesion DNA polymerase.^[^ ^29^, ^34^ ^]^ This suggests that DNA polymerase eta in human cells is especially proficient in preventing these UV‐induced C>A, T>A, and T>C substitutions (**Figure** **5**A), potentially because these non‐canonical UV mutations are more likely to be detrimental to protein function^[^ ^34^ ^]^ and cause driver mutations in oncogenes such as *BRAF* and *NRAS* in melanoma.^[^ ^2^, ^3^ ^]^ Yeast DNA polymerase eta, in contrast, is much less proficient at preventing non‐canonical UV mutation classes.^[^ ^32^ ^]^ Unlike human XPV/POLH, yeast Rad30 does not suppress UV‐induced T>A substitutions and actually causes many T>C substitutions (Figure 5B). Taken together, these findings suggest that similar mutational processes operate in both UV‐irradiated yeast and human cells, but the resulting mutation spectra are profoundly shaped by the differing activities of DNA polymerase eta (Figure 5). It will be important in future studies to elucidate the molecular mechanisms responsible for these species‐specific differences in polymerase eta activity and characterize the functions of DNA polymerase eta in other species.

A key question is why human DNA polymerase eta is less proficient at preventing UV‐induced C>T mutations, which dominate the mutation spectra of human skin cancers.^[^ ^1^, ^2^, ^3^, ^5^ ^]^ One possible explanation is that these C>T mutations may arise from cytosine‐containing CPD lesions in which the cytosine base has undergone accelerated deamination to uracil.^[^ ^67^, ^68^, ^69^ ^]^ Even error‐free bypass of a deaminated CPD by DNA polymerase eta would result in a C>T mutation, since it would correctly insert an adenine opposite the uracil base in the deaminated CPD.^[^ ^49^, ^53^ ^]^ Since human cells proliferate more slowly than yeast (i.e., >24 hours in human cells versus ∼1.5 hours in yeast), cytosine deamination in unrepaired CPDs may play a disproportionately more important role in UV mutagenesis,^[^ ^5^, ^70^, ^71^ ^]^ potentially contributing to the enrichment of C>T substitutions in human skin cancers.

One of the main surprises from studies investigating the role of DNA polymerase eta in UV mutagenesis is the unexpected appearance of TG>TT mutations in both human *XPV^−/−^* mutated skin cancers and *rad30*∆ yeast.^[^ ^32^, ^34^ ^]^ While this mutation class comprises 27% of all mutations in *XPV^−/−^* tumors, its underlying cause is still unclear. One potential mechanism for generating these mutations is indirect damage caused by UV‐induced ROS, resulting in 8‐oxoguanine lesions that are known to cause G>T substitutions.^[^ ^27^ ^]^ Alternatively, these lesions may be caused by a novel TG photoproduct,^[^ ^34^ ^]^ although the existence of this photoproduct has yet to be verified. It will be important in future studies to identify the specific DNA lesions responsible for this and other non‐canonical UV mutation classes.

Finally, while we have primarily focused on UV‐induced single base substitutions, UV also induces many tandem mutations and potentially other mutation classes, including insertion/deletion events (e.g.,^[^ ^26^ ^]^) and chromosomal copy number changes. It will be important in future studies to characterize the UV lesions potentially responsible for these different mutation classes, as well as the role of TLS DNA polymerases in these mutational processes. For example, a recent study indicates that yeast Rad30 prevents canonical CC>TT substitutions but causes non‐canonical AC>TT mutations.^[^ ^32^ ^]^ Additionally, while the mutation spectra induced by UVA and UVB light have been studied extensively, the effect of direct sunlight exposure on mutagenesis is less well characterized, but will be important to investigate in future genome sequencing studies.

## Conflict of Interest

The authors declare no conflict of interest.
